# Effect of Maternal Age and Fertility Treatment on the Increase in Multiple Births in Japan: Vital Statistics, 1974–2009

**DOI:** 10.2188/jea.JE20100189

**Published:** 2011-11-05

**Authors:** Syuichi Ooki

**Affiliations:** Department of Health Science, Ishikawa Prefectural Nursing University, Ishikawa, Japan

**Keywords:** multiple-birth rate, spontaneous multiple births, iatrogenic multiple births, maternal age, fertility treatment

## Abstract

**Background:**

The present study used vital-statistics data to estimate the effect of maternal age and fertility treatment on the number and rate of multiple live births in Japan from 1974 through 2009.

**Methods:**

Japanese vital statistics published by the Ministry of Health, Labour and Welfare from 1974 to 2009 were gathered and reanalyzed with regard to maternal age class and plurality of live births. The numbers of spontaneous and iatrogenic multiple births during 1977–2009 were estimated, with the assumption that spontaneous multiple-birth rates according to maternal age class would be constant and equal to those of baseline values, ie, the means between 1974 and 1976.

**Results:**

During the 25-year period, multiple-birth rates according to maternal age class increased after the late 1980s. This tendency was obvious in women aged 35 to 39 years. The estimated numbers of iatrogenic multiple births remained nearly constant in women aged 20 to 24 years and greatly increased in women aged 30 to 34 and 35 to 39 years. The rate (per 1000 live births) of iatrogenic multiple births gradually increased from 0.7 (1977) to 1.3 (1986), then rapidly and markedly increased from 1.3 (1986) to 11.4 (2005), and finally decreased to 8.1 (2009). The estimated maximum percentage of iatrogenic multiple births was 50.0%, in 2004 and 2005.

**Conclusions:**

The rapid increases in Japan in the number and rate of multiples born to women older than 30 years are likely due to iatrogenic rather than spontaneous multiple births.

## INTRODUCTION

Many factors affect spontaneous multiple-birth rates; the established factors are ethnicity, heredity, maternal age, and parity.^1^^–^^5^ These effects apply exclusively to dizygotic twinning. In addition, the occurrence of spontaneous multiple births increases with advancing maternal age up to approximately age 40, and with increased parity. However, this phenomenon has become more complicated in the majority of developed countries because this age range coincides with that of individuals who seek fertility treatment. Numerous studies have shown that increased use of fertility treatment, both assisted reproductive technologies (ART) and non-ART ovulation stimulation, as well as rising maternal age, have resulted in an increase in multiple births in developed counties worldwide.^6^^–^^14^

Secular trends in the multiple-birth rate in Japan have been described in detail elsewhere,^15^ and the present author will only briefly discuss them here. The multiple-birth rate is defined as the proportion of all live multiple births per 1000 live births. According to this definition, the multiple-birth rate in Japan was nearly constant between 1951 and 1976 (10.4–11.4). The values for these years in Japan can be regarded as spontaneous multiple-birth rates. The rate gradually increased to 12.4 in 1986, after which it rapidly increased and reached a peak in 2005 at 22.7, nearly double the spontaneous multiple-birth rate. The multiple-birth rate has gradually decreased over the past 4 years (2006–2009) to 19.5 (2009), which is likely due to the effect of the single-embryo transfer policy for ART.

These trends in multiple births are generally ascribed to fertility treatment and increased spontaneous dizygotic twinning in older mothers.^10^ Estimating the numbers or rates of iatrogenic singletons is very difficult because the total number of fertility treatments is generally unknown. However, it is possible to estimate the numbers or rates of iatrogenic multiples under several conditions, as discussed below. The purpose of this study was to separate the effects of fertility treatment from those of maternal age with regard to the multiple-birth rate during the last 30 years in Japan, a topic that has not been previously investigated in Japan.

## METHODS

### Dataset

All available vital statistics on multiple births in the entire Japanese population since 1974—originally collected by the Ministry of Health, Labour and Welfare—were gathered, combined, and reanalyzed. The vital statistics are based on birth records, which are published as an annual report of aggregate, not individual, data. Concerning maternal age, the number of all registered live births were obtained according to plurality (singletons or multiples) and maternal age strata, which were divided into 6 categories: younger than 20 years, 20 to 24, 25 to 29, 30 to 34, 35 to 39, and 40 years or older. Although the percentage of neonates born to mothers under 20 years and those 40 years or older were low (<1% and <3%, respectively), these data were also used in the statistical analyses.

### Statistic analyses

First, multiple-birth rates were analyzed according to maternal age class. Next, the number and rate of spontaneous and iatrogenic multiple births were estimated using the following method. The limited available data made it impossible to estimate directly the relative effect of maternal age and fertility treatment. However, the spontaneous multiple-birth rate according to maternal age is nearly constant irrespective of birth year.^1^^–^^5^ Thus, the number or proportion of naturally conceived multiple births in 1997–2009 can be estimated by multiplying the mean age-specific live multiple-birth rates between 1974–1976 by the age-specific total number of births, including both singletons and multiples, from the 1977–2009 data.

The mean live multiple-birth rates between 1974 and 1976 by maternal age class were used as the spontaneous live multiple-birth rates because the number of multiple births by fertility treatment was negligible during this period in Japan. The author used the weighted mean of 3 years to reduce the fluctuation that is present in the annual data.

By summing the numbers of all age classes, the total number of spontaneous multiple births can be estimated, after which spontaneous multiple-birth rates can be calculated. The number of iatrogenic multiple births was estimated by subtracting the spontaneous multiples from the total multiples, after which the multiple-birth rates could be calculated.

## RESULTS

In the following results, secular trends in mothers younger than 20 years and those older than 39 years were omitted because of excessive fluctuation in the data.

### Maternal age in Japan

**Figure 1.**
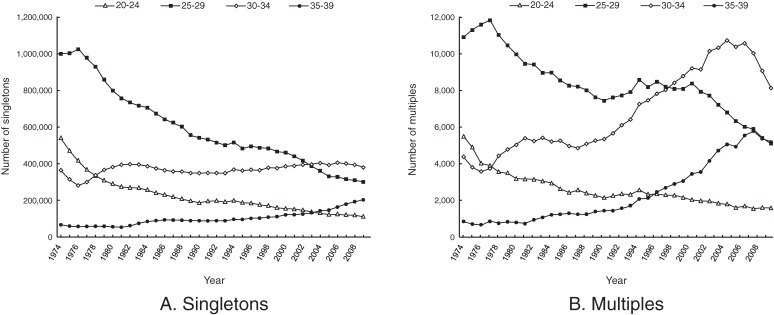
Secular trends in numbers of births by maternal age class, 1974–2009

Figure 1 shows the maternal age-class distribution by plurality. Regarding singletons, there is a marked decrease in births to mothers aged 25 to 29 years. In contrast, the number of singletons born to mothers aged 30 to 34 has remained relatively constant in recent years. Multiple births to women aged 30 to 34 and 35 to 39 are increasing, while those to women aged 20 to 24 and 25 to 29 are decreasing.

**Figure 2.**
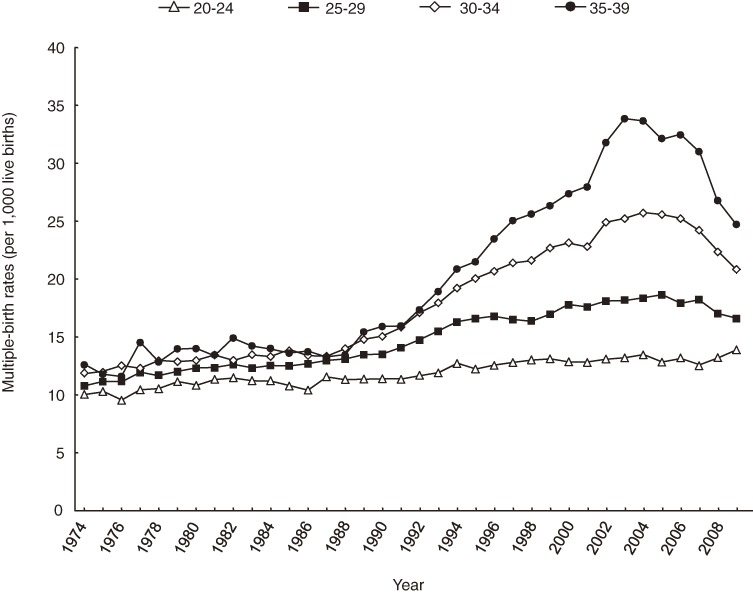
Secular trends in multiple-birth rates by maternal age, 1974–2009

Figure 2 shows multiple-birth rates by maternal age class. Multiple-birth rates remained constant until the mid-1980s, with slightly higher birth rates in older mothers. The difference by age in multiple-birth rates became greater after the mid-1980s. This tendency was most obvious in mothers aged 35 to 39.

Although not shown in Figure 2, the multiple-birth rates according to maternal age class from 1974–1976 were nearly the same for these 3 years and tended to increase linearly from age 20 to 24 to age 30 to 34 years, which supports the established observation mentioned in the Methods section for the present dataset. Thus, the present author used the weighted mean of the 3 years, ie, 8.7 per 1000 live births for those younger than 20, 9.9 for age 20 to 24, 11.0 for age 25 to 29, 12.1 for age 30 to 34, 12.0 for age 35 to 39, and 10.2 for age 40 years or older.

### Effect of maternal age on spontaneous and iatrogenic multiple births

**Figure 3.**
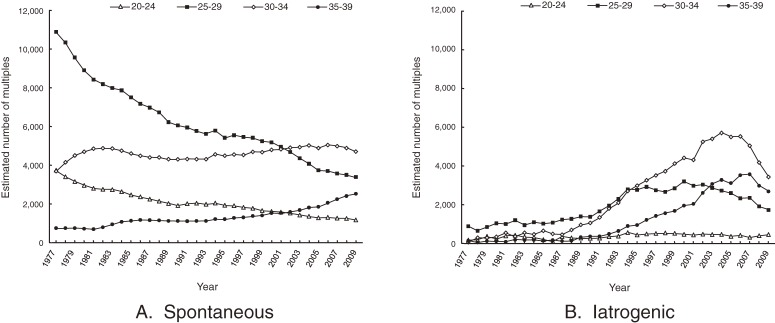
Estimated number of multiple births by method of conception and maternal age class (1977–2009). The number of naturally conceived multiple births in 1997–2009 was estimated by multiplying the mean age-specific multiple-birth rates between 1974–1976 with the age-specific number of births from 1977–2009. The number of iatrogenic multiple births (B) was calculated by subtracting the number of spontaneous multiples (A) from the number of total multiples (Figure 1B).

Figure 3 shows the estimated number of multiple births according to maternal age class and method of conception. The number of iatrogenic multiple births (B) was calculated by subtracting the number of spontaneous multiples (A) from the total number of multiples (Figure 1B). The number of spontaneous multiple births markedly decreased among mothers aged 25 to 29, but remained nearly constant for age 30 to 34. The number of iatrogenic multiple births markedly increased among mothers aged 30 to 34 and 35 to 39, but has recently decreased.

**Figure 4.**
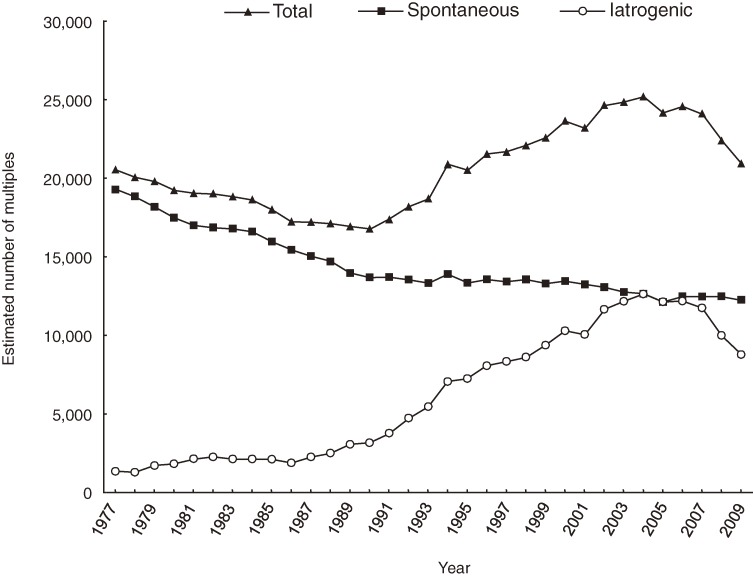
Secular trends in the sum of age-specific estimates of spontaneous and iatrogenic multiple births, 1977–2009

Figure 4 shows the secular trend in the sum of the age-specific estimated numbers of spontaneous and iatrogenic multiple births. The number of spontaneous multiple births decreased throughout the period, whereas the number of iatrogenic multiple births increased gradually until the mid-1980s, after which it increased rapidly. The estimated maximum percentage of iatrogenic multiple births among total multiple births was 50.0%, in 2004 and 2005, after which it decreased to 41.7% in 2009.

Regarding multiple-birth rates, spontaneous multiple-birth rates slightly increased from 11.0 (1977) to 11.4 (2009) because the distribution of maternal age shifted higher with year. However, iatrogenic multiple births gradually increased from 0.7 (1977) to 1.3 (1986), suddenly and markedly increased from 1.3 (1986) to 11.4 (2005), and then decreased to 8.1 (2009). This rapid increase in the multiple-birth rate after the mid-1980s was almost entirely due to iatrogenic multiple births.

## DISCUSSION

The estimated numbers of spontaneous multiple births shown in Figure 3A, which were calculated by multiplying the mean age-specific multiple-birth rates between 1974–1976 with the age-specific number of births from 1977–2009, closely reflect the secular trend in the numbers of singletons according to maternal age class, as shown in Figure 1A. This result also suggests that the effect of advanced maternal age on increased spontaneous multiple births is not large.

Although mothers aged 30 to 34 years have recently accounted for the largest proportion of multiple births, the actual number of spontaneous multiple births among this age class has remained nearly constant (Figure 3A). Therefore, the substantial change in the number of multiples among mothers aged 30 to 34 is mainly due to iatrogenic multiples (Figure 3B). Although the estimated number of spontaneous multiples born to women aged 35 to 39 has increased, the decrease in such births to women aged 25 to 29 is more dramatic (Figure 3A). This secular trend was similar to those of singletons, which gradually increased in women aged 35 to 39 and markedly decreased in women aged 25 to 29 (Figure 1A). The overall number of spontaneous multiple live births is decreasing (Figure 4).

Tandberg et al^10^ showed the contribution of maternal age in the increase in multiple births, even after excluding multiple births by ART. However, they did not consider fertility treatment with non-ART ovulation-stimulation drugs, the use of which also increases with maternal age. Most countries have no official data on non-ART ovulation stimulation,^14^ which presents a challenge for epidemiologic monitoring of iatrogenic births. The present study showed that secular trends in multiples in Japan are mainly attributable to iatrogenic rather than spontaneous multiples in older women, especially those aged 30 to 34, as shown in Figure 3B.

In a recent study, Schieve et al^14^ used US vital statistics and ART data to estimate the contribution of non-ART ovulation stimulation fertility treatment on multiple births in that country. They estimated that the percentage of iatrogenic multiple births in 2005 was 40.1%, which is approximately 10% lower than the present estimate for Japan in the same year (50.0%).

The approximation formulae used in the present study overestimate spontaneous multiple births^16^ because the maternal age distribution of all live births, both singletons and multiples, between 1977–2009 is assumed to be spontaneous, even though some are iatrogenic. In other words, the present results underestimate the numbers of iatrogenic multiples. Logically, iatrogenic infants should have been excluded from this calculation. Unfortunately, there is no other satisfactory method for estimating the numbers of iatrogenic multiples under the present circumstances in the absence of a complete registry of fertility treatment, especially for non-ART multiples.^16^

A limitation of the present study was that the present data could not be used to directly separate fertility treatment from confounding factors such as maternal age and parity.^1^ Parity is an independent factor that affects spontaneous dizygotic twinning.^1^ The dizygotic but not monozygotic twinning rate increases with parity. Regarding parity, the number of children born to each mother is rapidly decreasing in Japan because of complicated socioeconomic factors, eg, the later marriage of women and an increase in the proportion of working women. Recently, parity is decreasing in mothers of multiples in Japan. For example, in 1979 about 40% of mothers were estimated to have given birth to their multiples in the first delivery; that percentage was about 45% in 1989 and approximately 60% between 1999 and 2009. These estimates were made by using vital-statistics data on the numbers of live-birth multiples according to birth order as individual neonates. For example, the number of second deliveries was estimated by subtracting the number of second-born multiples from first-born multiples. Therefore, the effect of parity on spontaneous twinning seems to have weakened.

There are other factors that affect multiple-birth rates, particularly the rate of spontaneous dizygotic twinning.^4^^,^^17^^–^^19^ The effect of maternal physique has also been reported recently. Taller mothers and those with a high body mass index have a greater likelihood of dizygotic twinning. Smoking, contraceptive use, seasonality, and folic acid show less convincing associations with multiple births. The influence of these factors has not been analyzed using Japanese data, but their effects are small when compared with maternal age, as shown in studies conducted in other countries.^17^^–^^19^

Mothers of multiple-birth children are clearly older than mothers of singletons. For example, in 2005 the percentage of mothers aged 35 years or older who gave birth to multiples was 23%, as compared with 16% who gave birth to singletons; in 2009 the respective values were 28% and 22%. Moreover, the estimated maximum percentage of multiple births that were iatrogenic was 50.0%, in 2004 and 2005. Advanced age makes the physical, mental, and social burden of rearing 2 babies at once even greater. This situation could increase the risk of maternal problems such as postpartum depression, difficulties with child rearing, and, in the worst case, child abuse.^20^^,^^21^

In conclusion, the present study found that although the multiple-births rate has been decreasing since 2005, the overall rapid increase in the real number of multiple births and the multiple-birth rate during the past 30 years in Japan is mainly due to iatrogenic, not spontaneous, multiple births among older mothers. In other words, higher maternal age results in more iatrogenic multiples, not more spontaneous dizygotic twins. Because of the medical and social impact of multiple births,^15^^,^^22^^,^^23^ there is an urgent need for a system of hospital-based monitoring of fertility treatments and multiple births in Japan.
